# The Contact Ageing Effect on Fretting Damage of an Electro-Deposited Coating against an AISI52100 Steel Ball

**DOI:** 10.3390/ma9090754

**Published:** 2016-09-03

**Authors:** Kyungmok Kim, Joon Soo Ko

**Affiliations:** Department of Aerospace Engineering, School of Aerospace and Mechanical Engineering, Korea Aerospace University, 76 Hanggongdaehang-ro, Deogyang-gu, Goyang-si, Gyeonggi-do 412-791, Korea; kkim@kau.ac.kr

**Keywords:** frictional ageing, fretting, electro-deposited coating, friction

## Abstract

This article investigates the effect of contact ageing on fretting damage of an epoxy-based cathodic electro-deposited coating for use on automotive seat slide tracks (made of cold-rolled high strength steel). Static normal load was induced at the contact between the coating and an AISI52100 ball for a certain duration. It was identified that plastically deformed contact area increased logarithmically as a function of time when the contact was under static normal load. Fretting tests after various durations of static contact were conducted using a ball-on-flat plate apparatus. All fretting tests were halted when the friction coefficient reached a critical value of 0.5, indicating complete coating failure. The total number of fretting cycles to the critical friction coefficient was found to vary with the duration of static contact before fretting. It was identified that the number of cycles to the critical friction coefficient decreased with the increased duration of static contact. Meanwhile, the friction coefficient at steady-state sliding was not greatly affected by the duration of static contact before fretting. Finally, the relation between coating thickness after indentation creep and the number of cycles to the critical friction coefficient was found to be linear. Obtained results show that the duration of static contact before fretting has an influence on the fretting lifetime of an electro-deposited coating.

## 1. Introduction

If static contact is made between two solid bodies, interfacial bonding at asperity contact is strengthened over contact time and asperity creep is observed at a constant normal force. Contact growth, coalescence, and formation of new contacts are found to occur at the microscopic level, leading to the increase in actual contact area at the macroscopic level [1,2]. It was identified that the real area of contact grew logarithmically at constant normal force [3]. The strengthening of interfacial bonding at asperity contact led to an increase in the force of static friction, thereby increasing the coefficient of static friction over contact time; it was found that the force of static friction grew logarithmically with contact time in static contact [4,5,6]. 

If small-amplitude reciprocal sliding between two bodies exists (known as fretting), wear or cracking occurs at contact [7]. In order to reduce fretting damage and friction between mechanical components, various thin solid coatings including diamond-like carbon (DLC), a molybdenum disulfide (MoS_2_) coating, and an electro-deposited coating are used [8,9]. Fretting wear resistance of DLC coatings was evaluated in water-lubricated conditions [8]. Multi-layer dry lubricant coatings containing Mo and S were found to reduce fretting wear in the dovetail connection of an aero-engine [9]. An epoxy-based electro-deposited coating was applied onto the automotive seat slide rails to reduce friction and increase corrosion resistance [10,11]. The coating is typically in contact with a steel ball or a solid roller. Sliding or rolling between an electro-deposited coating and a ball occurs while an automotive seat is adjusted. When the seat is fixed, normal force merely exists at the contact surface between an electro-deposited coating and a ball. The electro-deposited coating is progressively damaged over time, since normal force remains at the contact between the coating and the ball. Thus, creep of the coating or frictional ageing may occur in the contact. Creep behavior of epoxy resins was studied [12,13]; the creep of an epoxy resin cured at atmospheric and hydrostatic pressure was identified under pure tension loading. However, little else was found in studies of compressive creep and frictional ageing of an epoxy-based electro-deposited coating. 

Fretting is an important issue in the evaluation of an electro-deposited coating for use on automotive seat slide tracks. Fretting wear of an electro-deposited coating was studied in terms of the kinetic friction coefficient [14]. Friction coefficient evolutions of electro-deposited coatings were evaluated against various counterparts. It is highly important to determine the friction coefficient evolution of the coating under fretting condition, since the performance of the coating under fretting was often taken into account as the number of fretting cycles. However, in the previous studies, the effect of contact ageing was not considered in evaluation of an electro-deposited coating. In order to obtain an accurate fretting lifetime of an electro-deposited coating, it is necessary to investigate the influence of the duration of static contact before fretting on a friction coefficient evolution. 

In this study, actual contact size on an electro-deposited coating was determined after various durations of static contact at constant normal force before fretting. Contact growth was then described with an appropriate mathematic form. In order to identify the effect of duration of static contact on fretting behavior of an electro-deposited coating, fretting tests were conducted after maintaining static contact between a ball and an electro-deposited coating for different durations. The evolution of the kinetic friction coefficient was determined and evaluated. Finally, the relation between the duration of static contact and fretting lifetime of an electro-deposited coating was identified.

## 2. Experimental Set-Up

### 2.1. Fretting Test Rig

**Figure 1:**
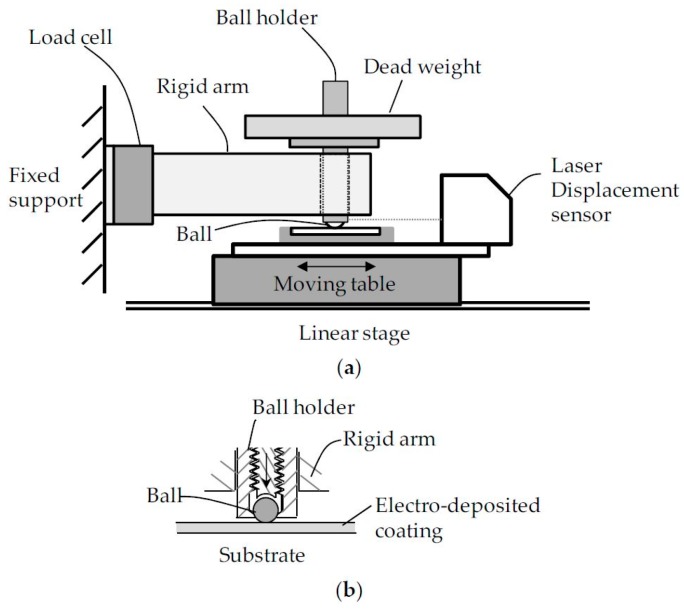
Schematic illustration of the test apparatus: (**a**) the side view of a fretting test rig; (**b**) ball-on-flat contact geometry.

Figure 1 illustrates a fretting testing machine using ball-on-flat contact geometry. The test machine is comprised of a linear stage, a rigid arm, dead weight, a ball holder, a load cell, and a laser displacement sensor. For a fretting test, one ball and one flat specimen were used. A flat specimen was fixed on the moving table of a linear stage (PImiCos GmbH, LS-110, Eschbach, Germany). A laser displacement sensor with a resolution of 0.003 mm and a linearity of ±0.1% (Keyence LK-081, Itasca, IL, USA) was located on the moving table. The laser displacement sensor measured the relative displacement between a flat specimen and a ball holder during each test. A Φ5 mm ball was gripped by a screw bolt in a ball holder. A ball holder was permitted to move vertically in a rigid arm. Dead weight was placed on the ball holder, inducing normal force to the contact between a ball and a flat specimen. During each test, normal force was maintained as constant. The rigid arm was connected to a load cell attached on the fixed support. 

**Figure 2:**
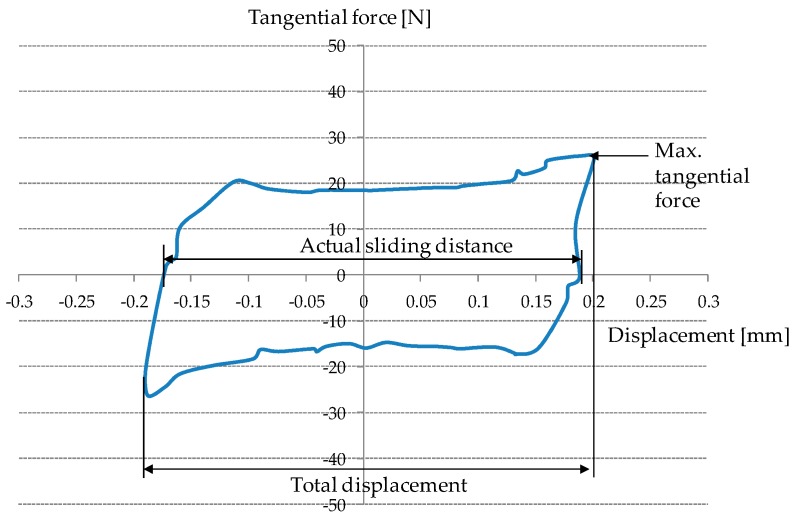
Fretting loop and definition of the maximum tangential force and sliding distance.

During each test, a tangential force and a relative displacement were recorded. A fretting loop was drawn after each cycle as presented in Figure 2. The maximum tangential force and actual sliding distance were then determined on a fretting loop. The kinetic friction coefficient was calculated as the ratio of the maximum frictional force to the imposed normal force. When the friction coefficient reached 0.5, a test was terminated. It was identified from the literature that the friction coefficient of 0.5 was close to the measured one between an AISI52100 ball and the substrate (cold-rolled high strength steel) [11].

### 2.2. Test Material and Test Condition

A cathodic electro-deposited coating was applied onto a cold-rolled high strength steel plate. The coating was based on an epoxy resin including a metal catalyst and a cross-linker of blocked aromatic isocyanates. Deposition condition of the coating was described in Table 1. Initial thickness of a coating layer was 0.02–0.03 mm. The coating maintained a Pencil hardness of 5 H. A commercial AISI52100 ball with a diameter of 5 mm was selected as a counterpart of the coating; the ball is being used for automotive seat slide tracks. The commercial AISI52100 ball maintained the arithmetic average surface roughness (*R*_a_) of 0.03 µm, an elastic modulus of 190–210 GPa, and *Poisson*’s ratio of 0.3, respectively. The hardness of AISI52100 balls was 60–63 HRC. Meanwhile, the substrate (cold-rolled high strength steel) maintained an elastic modulus of 205 GPa and *Poisson*’s ratio of 0.28. 

Before fretting testing, static contact between an electro-deposited coating and a ball was made for durations of 1, 10^3^, 10^5^, and 5 × 10^5^ s. During static contact, a normal force was maintained as constant. In order to induce fretting similar to those found on automotive seat slide tracks, a normal force of 49 N was applied to the contact between a ball and a flat specimen; for determining the magnitude of normal force, it was assumed that a loaded seat weighed 80 kg and contained 16 balls. After applying a normal force of 49 N, the displacement amplitude of 0.2 mm was induced with a frequency of 1 Hz. All tests were performed at room temperature of 22–24 °C and relative humidity of 50%–60%.

## 3. Results and Discussion

### 3.1. Determination of Plastically Deformed Contact Region

**Figure 3:**
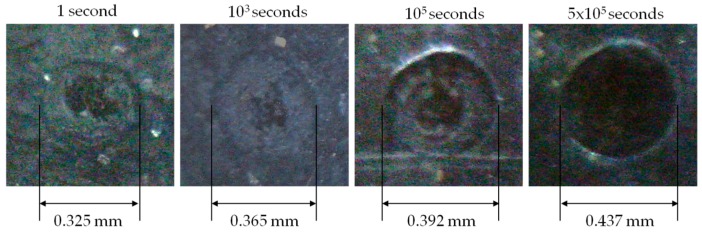
Image of a plastically deformed region on an electro-deposited coating after different durations of static contact at a normal force of 49 N.

**Figure 4:**
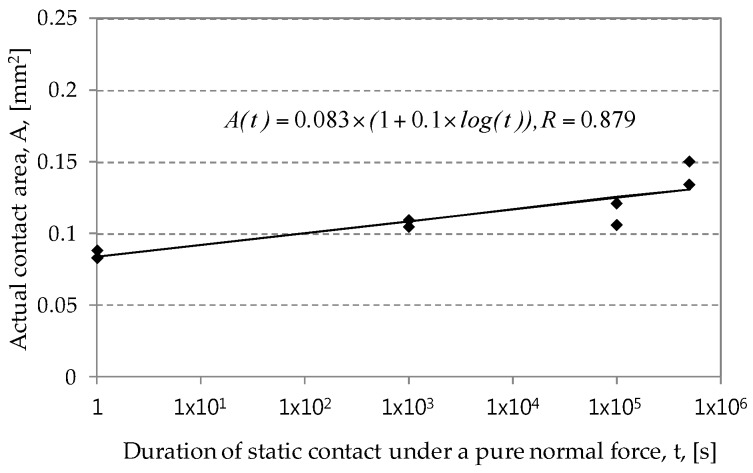
Actual contact area as a function of duration of static contact at a normal force of 49 N. A smooth line denotes a fitted curve.

A normal force of 49 N was induced at the contact surface between an AISI52100 ball and an electro-deposited coating. Normal loading was then maintained for 1, 10^3^, 10^5^, and 5 × 10^5^ s. After removing a ball from the contact, the contact surface of an electro-deposited coating was observed. Figure 3 shows damaged surfaces after various durations of static contact under pure normal force. The surface image after 1 s shows that a normal force of 49 N led to plastic deformation (spherical indentation) on the surface of an electro-deposited coating. Actual contact size was measured with a microscope. The diameter of a plastically deformed region was 0.325 mm after 1 s. The contact region was found to enlarge as the duration of static contact increased. After 5 × 10^5^ s, a plastically deformed region was 0.437 mm in diameter. Figure 4 shows the growth of actual contact area with respect to duration of static contact. As the applied normal force was constant, the average value of a compressive stress at contact surface was calculated as 572 MPa after 1 s and 344 MPa after 5 × 10^5^ s. 

It is possible to describe the growth of actual contact area with an indentation creep model proposed by Schloz and Engelder [3]. In the model, actual contact area increases during the penetration of asperities of the hard material through the soft surface. That is, actual contact area is expressed as a function of the duration of static contact.

(1) $$A(t)=\left(1+c\times \log(t)\right)\frac{P}{H},$$

where *P* is normal force, *H* is the hardness of soft material measured at unit time, *t* is duration of static contact under a constant normal force, and *c* is the nondimensional coefficient. The coefficient *c* is associated with the growth of actual contact area. 

If the hard material is spherical, Equation (1) can be rewritten with contact radius *a*.

(2) $$a(t)=\sqrt{\frac{P}{\pi H}(1+c\times \log(t))}.$$

**Figure 5:**
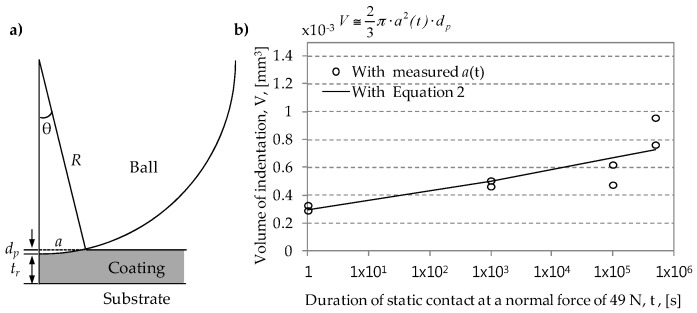
Determination of indented volume: (**a**) schematic diagram of static contact between a hard ball and a soft coating; (**b**) estimated volume of indentation after unloading. *d_p_* denotes the penetration displacement.

When a hard ball and a soft coating are placed in contact under a constant normal force, a ball gradually penetrates the soft coating layer through indentation creep, as shown in Figure 5a. Volume of indentation during coating creep can be approximately calculated as Figure 5b (an indented shape was assumed to be semi-ellipsoidal). A coating thickness *t_r_* remained after coating creep can then be determined as

(3) $$t_{r}=t_{0}-R(1-\cos(\sin^{-1}(\frac{a}{R})),$$

where *t_0_* is initial coating thickness before static contact, and *R* is the radius of a ball.

The coating thickness after creep is expressed as a function of contact radius *a*. The coating thickness after creep is of interest, since it could affect the fretting lifetime of a coating.

### 3.2. Fretting Wear Test Results

Fretting wear tests using ball-on-flat apparatus were then conducted for investigating the effect of duration of static contact on the kinetic friction coefficient evolution of an electro-deposited coating. After inducing a normal force of 49 N for various durations of static contact, the displacement amplitude of 0.2 mm was induced at the contact surface between a ball and a coating with a frequency of 1 Hz. In the previous studies [15], the displacement amplitude of 0.2 mm at a normal force of 49 N led to gross slip at the contact surface between an AISI52100 ball and an electro-deposited coating. Tests were terminated when the friction coefficient became 0.5. All tests were conducted twice at the same test condition.

**Figure 6:**
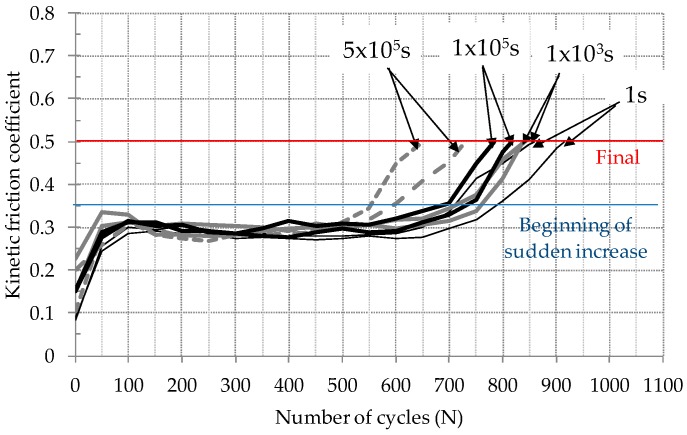
Friction coefficient evolutions of electro-deposited coatings against AISI52100 balls after various durations of static contact.

Figure 6 shows the kinetic friction coefficient evolution of an electro-deposited coating against an AISI52100 ball. Fretting tests were started after inducing normal force for predefined durations of static contact. The kinetic friction coefficient was determined as the ratio of the maximum tangential force to normal force. The initial friction coefficient remained below 0.2 except the coefficient for 10^3^ s (Test 2). All friction coefficients became stable, following the initial increase up to 100 cycles (so-called initial running-in period). Table 2 shows the friction coefficient during a stable stage (called as steady-state sliding). Without regard to the duration of static contact before fretting, the friction coefficient ranged from 0.29 to 0.3. No correlation between duration of static contact and the friction coefficient was found. After a stable stage, the friction coefficient increased dramatically.

**Figure 7:**
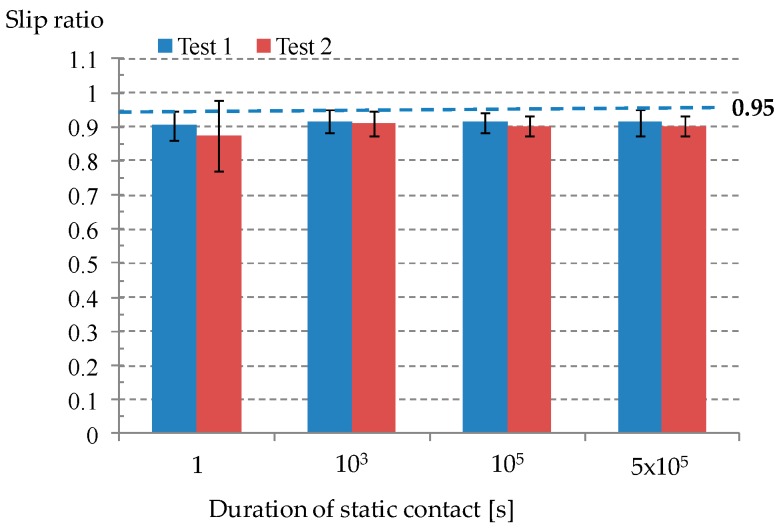
The average slip ratio. A slip ratio of 0.95 indicates the transition from a gross slip regime to a reciprocal sliding regime.

During a fretting wear test, a slip regime needs to be identified. In this study, slip ratio was used for ensuring that all tests were completed within a gross slip regime. Slip ratio was defined as the ratio of actual sliding distance to imposed total displacement. A slip ratio of 0.95 indicates the transition from a gross slip regime and a reciprocal sliding regime [16]. Figure 7 shows that all average values of slip ratio remained below 0.95.

**Figure 8:**
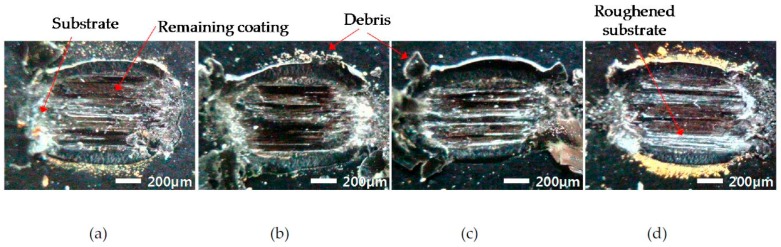
Captured surface images at the friction coefficient of 0.5: (**a**) 1 s; (**b**) 10^3^ s; (**c**) 10^5^ s and (**d**) 5 × 10^5^ s.

At a friction coefficient of 0.5, fretted surfaces of an electro-deposited coating were captured with a microscope. Figure 8 shows the damaged surfaces for various durations of static contact at a normal force of 49 N. All images show that the contact surfaces were severely damaged; near the left and right side of the contact, a hard ball penetrated a soft coating layer and the substrate appeared. In the middle of contact, some parts of a coating were observed to remain. The ball generated strong scratches on the surface of the coating along the sliding direction. In addition, the roughened substrate was partially found in the middle of contact. Debris of a coating was observed in the vicinity of a contact region. 

**Figure 9:**
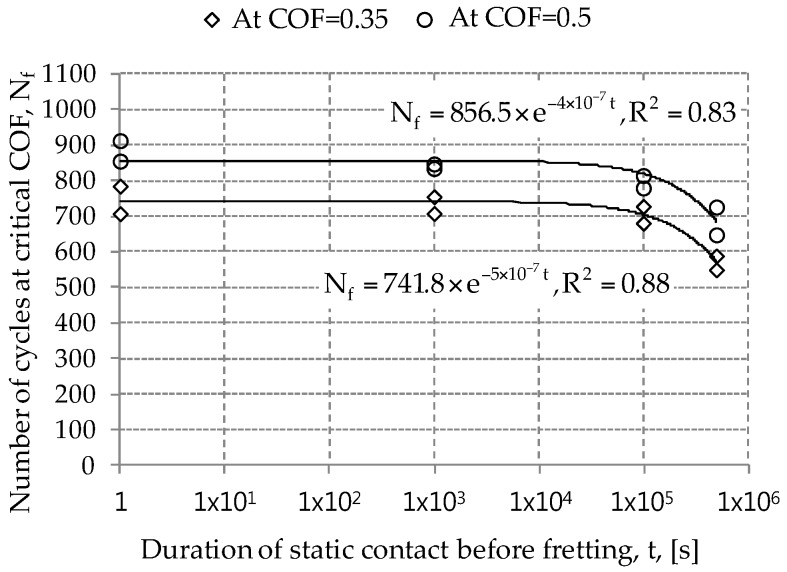
Number of cycles to the critical friction coefficient (COF) versus the logarithm of the duration of static contact before fretting. Smooth lines are fitted curves. The COF of 0.35 presents the beginning of sudden increase after a steady state sliding, while the value of 0.5 indicates the final of the test.

The fretting lifetime of a solid coating was often determined on the friction coefficient evolution [17,18]. That is, the fretting lifetime of a solid coating was defined as the number of cycles to the critical friction coefficient. For solid coatings, the critical friction coefficient was considered as 0.5. In this study, the fretting lifetime of an electro-deposited coating was determined on the kinetic friction coefficient presented in Figure 6. Two friction coefficients of 0.35 and 0.5 were selected as a critical value. The critical value of 0.35 presents the beginning of a sudden increase on the friction coefficient after a steady state sliding, while the value of 0.5 indicates the end of the test. Figure 9 shows the relation between the total number of cycles to the critical friction coefficient and duration of static contact before fretting. It was observed that the fretting lifetime of the electro-deposited coating decreased with the increased duration of static contact before fretting. Fretting lifetime of the electro-deposited coating after 5 × 10^5^ s was found to be 22% lower than that after 1 s (for the critical friction coefficient of 0.5). In Figure 9, the relation between the critical number of fretting cycles and the duration of static contact before fretting was obtained by employing curve fitting. In further work, the relation needs to be derived from a physical law.

**Figure 10:**
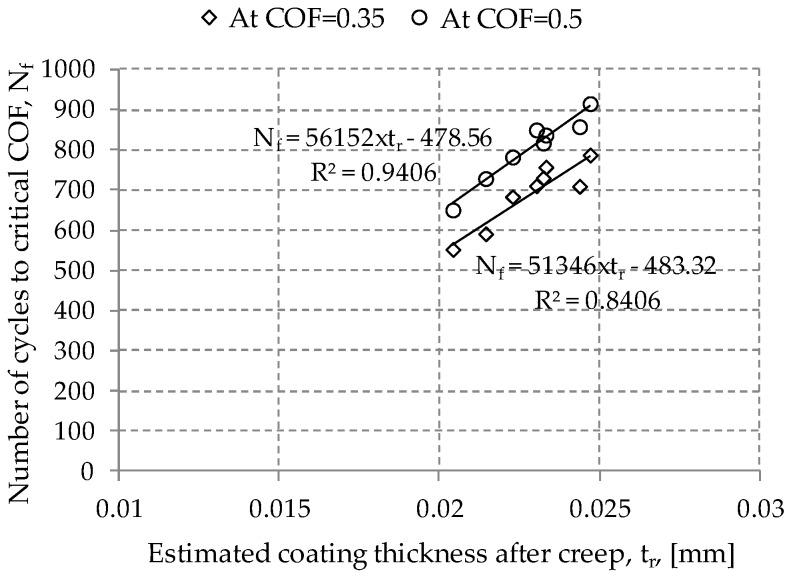
Relation between number of cycles to the critical friction coefficient (COF) and the estimated coating thickness after indentation creep. Smooth lines are fitted curves. The COF of 0.35 presents the beginning of sudden increase after a steady state sliding, while the value of 0.5 indicates the final of the test.

Figure 10 shows the relation between fretting lifetime of an electro-deposited coating and coating thickness remained after indentation creep. It was found that fretting lifetime increased as the coating thickness after creep increased. The relation between the fretting lifetime and the coating thickness after creep can be expressed as a linear function. It can be identified from Figure 9 and Figure 10 that plastic deformation resulting from coating creep brought about the reduction of the fretting lifetime of an electro-deposited coating.

In this study, an AISI52100 ball as the counterpart of an electro-deposited coating was selected. Thus, further tests need to be conducted with other metallic and ceramic balls. In addition, the relation between fretting lifetime and duration of static contact before fretting needs to be described with an appropriate law. In this study, approximate volume of indentation was calculated. Thus, actual cross-sectional area on an indented surface needs to be measured. In Figure 6, the maximum tangential force in a fretting loop was selected for determining a friction coefficient evolution. Meanwhile, the average of positive (or negative) tangential forces needs to be considered for identifying frictional energy dissipation, since accumulated dissipated frictional energy can be used for estimating the durability of a solid coating. 

## 4. Conclusions

The following conclusions were drawn.

- Frictional contact between an AISI52100 ball and an electro-deposited coating led to plastic deformation on an electro-deposited coating under pure normal force. Spherical indentation was found to remain on the coating after unloading. The plastically deformed contact region increased with the increased duration of static contact under a constant normal force. An actual contact area was found to be expressed as a logarithmic function.
- Fretting test results with various durations of static contact before fretting showed that the kinetic friction coefficient values at the initial and steady-state sliding stages were not affected by the duration of static contact before fretting.
- Number of fretting cycles to the critical friction coefficient (i.e. 0.35 and 0.5) was found to decrease with the increased duration of static contact before fretting. It can be concluded that creep of an electro-deposited coating leads to degradation of fretting resistance of an electro-deposited coating.

Further work should include an investigation on the apparent relation between fretting lifetime and the duration of static contact before fretting. The mathematical description of the relation remains a challenging work. In this study, the approximate volume of indentation was calculated. Thus, the actual cross-sectional area on an indented surface needs to be measured. 

## Figures and Tables

**Table 1:** Deposition condition of an electro-deposited coating.

Specification	Magnitude
Solid (%)	15–22
Pigment binder ratio	~0.15
pH	5.9–6.3
Conductivity (µS/mm)	120–180
Deposition time (s)	~180

**Table 2:** The kinetic friction coefficient (COF) between an electro-deposited coating and an AISI52100 ball.

Duration of Static Contact (s)	Initial COF		Steady COF Mean ± 6 × Standard Deviation	
	Test 1	Test 2	Test 1	Test 2
1	0.084	0.089	0.292 ± 0.074	0.283 ± 0.042
10^3^	0.228	0.152	0.296 ± 0.065	0.304 ± 0.027
10^5^	0.152	0.150	0.292 ± 0.050	0.307 ± 0.064
5 × 10^5^	0.201	0.284	0.290 ± 0.049	0.284 ± 0.050
